# ASSOCIATION BETWEEN SEEKING HEALTH INFORMATION ON THE INTERNET AND PALLIATIVE CARE KNOWLEDGE

**DOI:** 10.1093/geroni/igz038.978

**Published:** 2019-11-08

**Authors:** YuJun Zhu, Susan Enguidanos

**Affiliations:** University of Southern California, Los Angeles, California, United States

## Abstract

Background: National and state surveys have found that only 8% - 17% of people know what palliative care (PC) is. Anecdotal reports also suggest many of those knowledgeable have misconceptions about PC. This lack of knowledge or misconceptions about PC can serve as barriers to accessing palliative care for seriously ill patients. Additionally, individuals often use the internet to seek health information, but it is unclear if this improves palliative care health literacy. Methods: We used the Health Information National Trends Survey 5, Cycle 2 (2018), a nationally representative dataset (n=3,504) to compare self-rated PC knowledge level with actual knowledge. We conducted chi-square and logistic regression to examine the association between PC knowledge and seeking health information on the internet. Results: About 33% of participants self-reported having PC knowledge. When these individuals were queried about specific PC knowledge, 58% failed to answer three basic questions correctly. After controlling for demographic and socioeconomic status, using the Internet to seek health information was positively associated with being knowledge about PC (P=0.006, OR=1.64), while having less than high school education was associated with lower PC knowledge. Implications: We found that self-reported PC knowledge may not reflect actual PC knowledge and thus clinicians should be sure to define the service when introducing it to patients. In addition, since use of internet in seeking health information is significantly associated with PC knowledge, it is important to ensure that internet PC information is valid, reliable, easy to access, and presented in simple language.

